# Crystal structure of (1*S*,2*R*,6*R*,7*R*,8*S*,12*S*)-4,10,17-triphenyl-15-thia-4,10-diaza­penta­cyclo[5.5.5.0^1,16^.0^2,6^.0^8,12^]hepta­deca-13,16-diene-3,5,9,11-tetrone *p*-xylene hemisolvate

**DOI:** 10.1107/S1600536814025094

**Published:** 2014-11-21

**Authors:** Wayland E. Noland, Neil J. Kroll, Matthew P. Huisenga, Ruixian A. Yue, Simon B. Lang, Nathan D. Klein, Kenneth J. Tritch

**Affiliations:** a Department of Chemistry, University of Minnesota, Minneapolis, MN 55455-0431, USA

**Keywords:** crystal structure, cyclo­addition, autoxidation, double addition, thio­phene

## Abstract

The title tetrone compound, C_32_H_22_N_2_O_4_S· 0.5C_8_H_10_, is the major product (50% yield) of an attempted Diels–Alder reaction of 2-(α-styr­yl)thio­phene with *N*-phenyl­male­imide (2 equivalents) in toluene. Recrystallization of the resulting powder from *p*-xylene gave the title hemisolvate; the *p*-xylene mol­ecule is located about an inversion center. In the crystal, the primary tetrone contacts are between a carbonyl O atom and the four flagpole H atoms of the bi­cyclo­[2.2.2]octene core, forming chains along [001].

## Abstract 2

A novel *meso* bi­cyclo­[2.2.2]octene-based compound was obtained from an attempted Diels–Alder reaction. It crystallizes from *p*-xylene as a hemisolvate.

## Chemical context   

**Figure 1:**
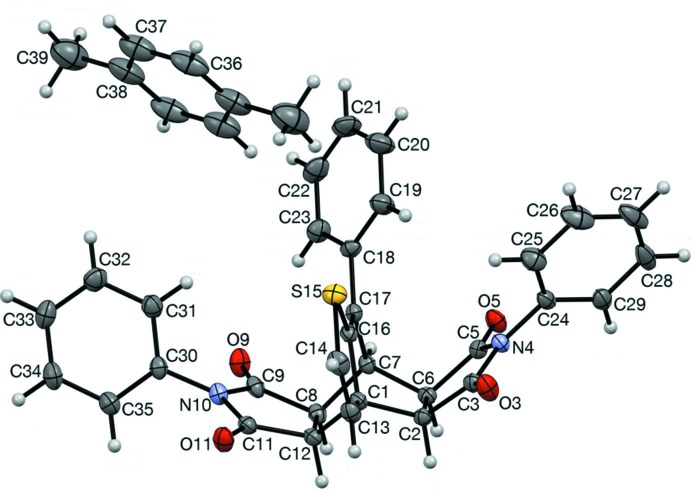
The mol­ecular structure of compound (3), with atom labelling (non-labelled atoms in the *p*-xylene solvent mol­ecule are related to the labelled atoms by inversion symmetry). Displacement ellipsoids are drawn at the 50% probability level.

The title compound, (3), is the first reported double-Diels–Alder adduct obtained from a one-pot reaction of a 2-vinyl­thio­phene (Fig. 1 ▶). This methodology may have use in the synthesis of novel ligands, zeolites, or polyamides.
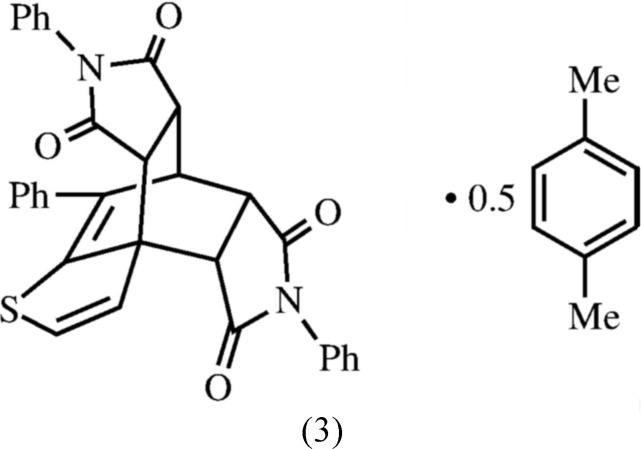


**Figure 2:**
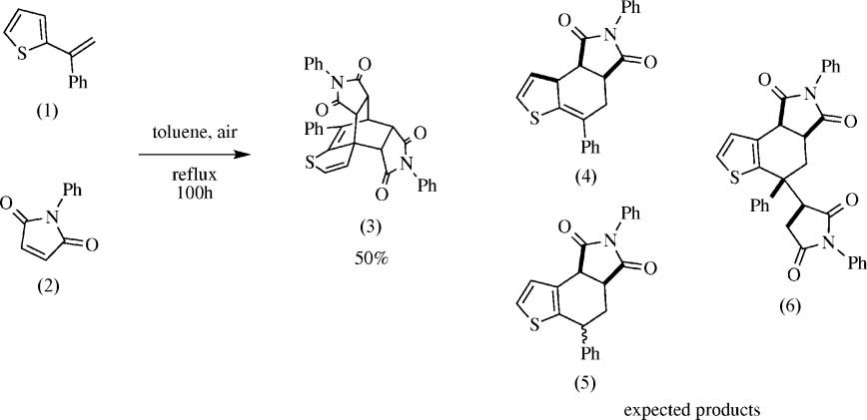
Synthesis of the title compound (3). Structures (4)–(6) were the expected products.

*Diels–Alder methodology:* Reactions between vinyl­heterocycles and dienophiles have been useful in natural product synthesis and in the development of potential medicinal compounds (Booth *et al.*, 2005 ▶; Kanai *et al.*, 2005 ▶). Reported heterocycles include indole, pyrrole (Le Strat *et al.*, 2005 ▶; Noland *et al.*, 2013 ▶), furan (Brewer *et al.*, 1971 ▶; Brewer & Elix, 1975*b* ▶; Davidson & Elix, 1970 ▶), benzo­furan, and benzo­thio­phene (Marrocchi *et al.*, 2001 ▶; Pihera *et al.*, 1999 ▶). A Diels–Alder reaction was attempted between 2-(α-styr­yl)thio­phene (1) (Tasch *et al.*, 2013 ▶) and *N*-phenyl­male­imide (2) in an effort to expand this methodology (Fig. 2 ▶). Based on work by Watson (2012 ▶), the expected products were adduct (4), aromatized adduct (5), or (6) *via* ene addition of (2) to (4). Given the scope of simpler products from these reactions, it was surprising to obtain tetrone (3) in such a high yield.

**Figure 3:**
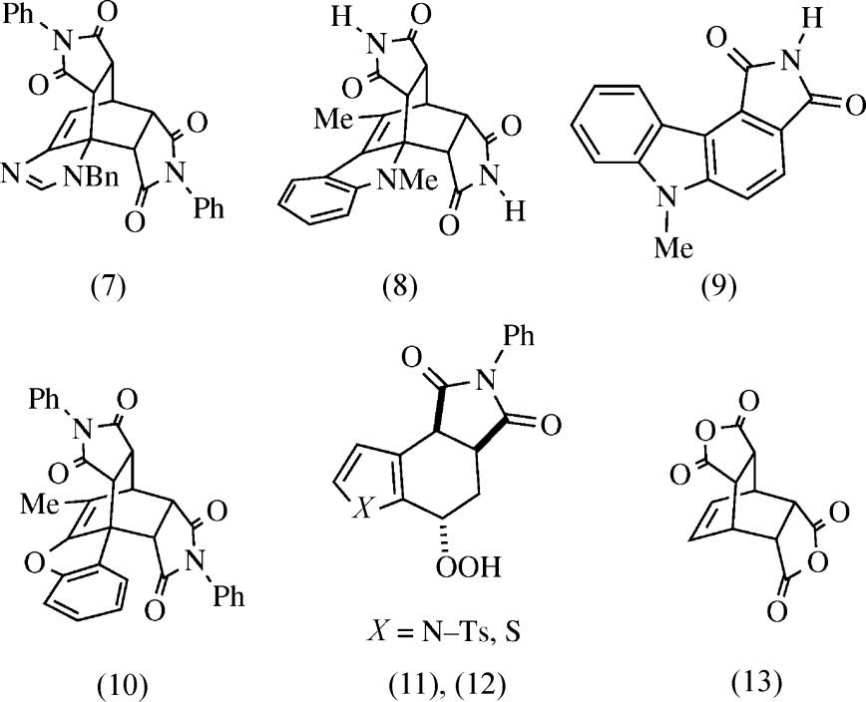
Contextual compounds. Double adducts (7) and (8) were previously reported. In acid, aromatized adduct (9) was favored over double addition. Double adduct (10) is the closest reported kin of (3). Recently reported (11) supports the proposed mechanism. Hydro­peroxide (12) is a likely inter­mediate to (3). Dianhydride (13) is commonly used for ligand synthesis.

*Mechanism:* Mechanisms proposed for double adducts (7) (Lovely *et al.*, 2007 ▶) and (8) (Noland *et al.*, 1993 ▶) suggest a Diels–Alder reaction (Fig. 3 ▶), with loss of H_2_ by an unknown pathway, and then a second cyclo­addition. Noland *et al.* (1993 ▶) observed that formation of (8) was accelerated by exposure to oxygen, and aromatization to (9) was favored over (8) in acid. Brewer & Elix (1975*a* ▶) reported a double adduct (10) and a hydro­per­oxy inter­mediate thereof; they proposed loss of H_2_ in an autoxidation followed by elimination of H_2_O_2_, a pathway that fits both observations made by the Noland group. The crystal structures of (3) and the hydro­peroxide (11) (Noland *et al.*, 2014 ▶), and preliminary HRMS and ^1^H NMR evidence that (12) is an inter­mediate to (3), all support the mechanism proposed by Brewer & Elix (1975*a* ▶).

*Applications:* Compounds related to (3) are used as bridging ligands in organometallic complexes (see: §4. *Database survey*), synthesis of zeolites (Cantín *et al.*, 2006 ▶; Inagaki *et al.*, 2013 ▶), and polyamides (Faghihi & Shabanian, 2010 ▶). Most examples are derived from dianhydride (13) (Hu, 2008 ▶) or a similar substrate, reacting with ammonia or primary amines, limiting variability to imido substitution. Domino method­ology has been developed that could give more diverse functionality (Strübing *et al.*, 2005 ▶).

## Structural commentary   

**Figure 4:**
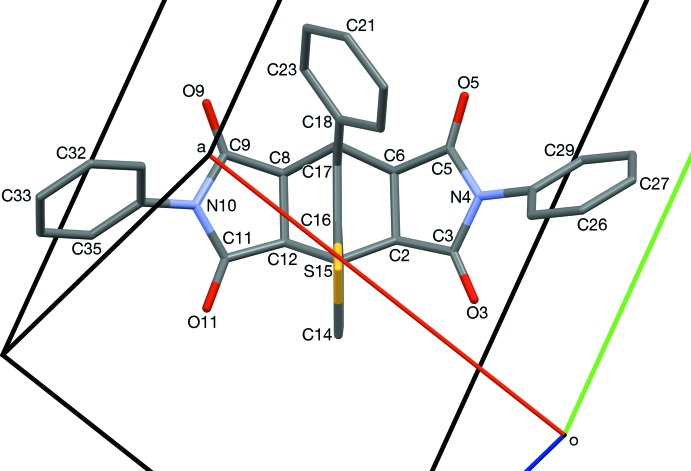
A mol­ecule of (3) viewed near [1
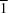
4], normal to the pyrrolo­[3,4-*g*]iso­indole ring system. The styryl­thio­phene unit (C21, C18, C17, C16, S15, C14) is forward. The *N*-phenyl rings are twisted so C26 and C32 are forward, C29 and C35 are behind.

In compound (3) (Fig, 1), the *N*-phenyl rings (C24–C29) and (C30–C35) are twisted out of the plane of their respective succinimido rings, (N4/C3/C2/C6/C5) and (N10/C9/C8/C12/C11), by 54.83 (8) and 54.97 (8)°, respectively, with the same chirality, giving helical character along the major axis (C27 to C33). Figs. 4 ▶ and 5 ▶ show a left-handed mol­ecule. The bi­cyclo­[2.2.2]octene rings have a typical boat shape. The other rings are nearly planar; the r.m.s. deviations from their respective mean planes are 0.026 and 0.030 Å for the succin­imido rings (N4/C3/C2/C6/C5) and (N10/C9/C8/C12/C11), respectively, and 0.01 Å for the 3-hydro­thieno ring (S15/C16/C1/C13/C14). The two succinimido rings are inclined to one another by 29.24 (8)° and the N-phenyl rings are inclined to one another by 54.55 (8)°. The phenyl ring (C18–23) is inclined to the the *N*-phenyl rings, (C24–C29) and (C30–C35), by 89.89 (8) and 64.82 (8)°, respectively. There is an intra­molecular C—H⋯O hydrogen bond present (Table 1 ▶).

## Supra­molecular features   

**Figure 6:**
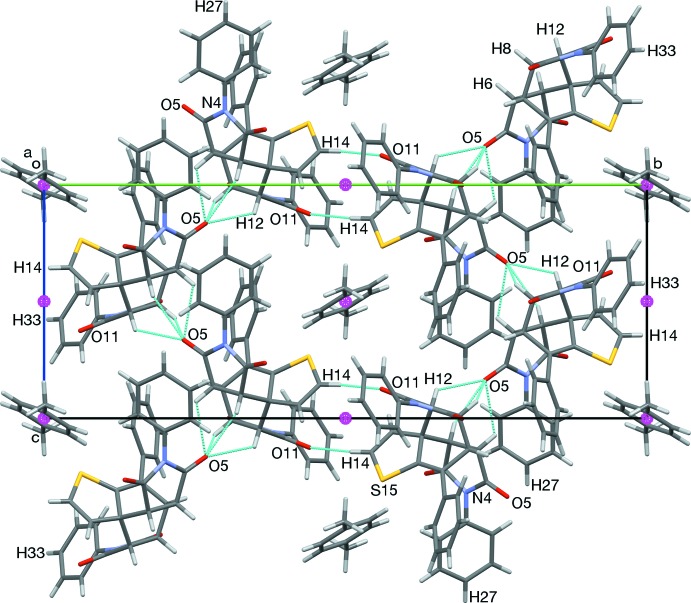
The crystal packing of compound (3) viewed along [100]. Chains of O5⋯H_*endo*_ hydrogen bonds form along [001]. *p*-Xylene and inversion-related pairs (O11⋯H14) of mol­ecules form a checker-board pattern.

In the crystal of (3), the carbonyl atom O5 forms weak hydrogen bonds with the *endo* face of the bi­cyclo­[2.2.2]octene unit, contacting H2, H6, H8, and H12. These contacts form chains along [001] (see Figs. 6 ▶ and 7 ▶, and Table 1 ▶). Weak O11⋯H14 hydrogen bonds form inversion dimers (Table 1 ▶).

## Database survey   

**Figure 8:**
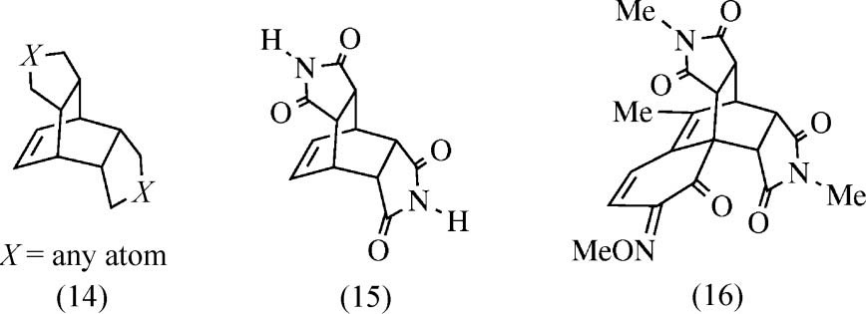
Selected database survey entries: substructure (14) was the basis of the survey. The di­imide (15) has been reported several times as a ligand. The coumarin-derived double adduct (16) is the only entry that is *spiro*-fused to a six-membered ring.

A search of the Cambridge Structural Database (Version 5.35, Update November 2013; Groom & Allen, 2014 ▶) was performed for *meso* structures derived from the parent structure (14); see Fig. 8 ▶. Fifteen organometallic entries were found, including inter­penetrating nets (Zhang *et al.*, 2011 ▶), container complexes (Liu *et al.*, 2007 ▶), and other multi-metal-center complexes (Yu *et al.*, 2012 ▶; Zhang, 2012 ▶). Thirteen organic entries were found, including the aforementioned (7), (8), and (11); an ammonia derivative (15) used as a ligand for inter­penetrating nets (Song *et al.*, 2012 ▶); and a coumarin-derived double-Diels–Alder adduct (16) (Nicolaides *et al.*, 1997 ▶).

## Synthesis and crystallization   

2-(α-Styr­yl)thio­phene (200 mg, Tasch *et al.*, 2013 ▶) and *N*-phenyl­male­imide (372 mg, 2 equiv.) were partially dissolved in toluene (5 mL). The resulting mixture was refluxed open to air for 100 h. Upon cooling to room temperature, the resulting suspension was separated by column chromatography (SiO_2_, hexa­ne:ethyl acetate, gradient from 1:0 to 1:1). The desired fraction (*R_f_* = 0.09 in 1:1) was concentrated at reduced pressure giving compound (3) as a white powder (287 mg, 50%, m.p. 554–555 K). ^1^H NMR (500 MHz, CD_2_Cl_2_) δ 7.498 (*dd*, *J* = 8.0, 1.5 Hz, 2H, H19, H23), 7.388 (*tt*, *J* = 7.0, 2.5 Hz, 4H, H26, H28, H32, H34), 7.374 (*td*, *J* = 5.0, 1.5 Hz, 2H, H20, H22), 7.351 (*tt*, *J* = 7.0, 1.5 Hz, 2H, H27, H33), 7.263 (*tt*, *J* = 4.5, 1.5 Hz, 1H, H21), 6.987 (*dd*, *J* = 7.0, 1.5 Hz, 4H, H25, H29, H31, H35), 6.600 (*d*, *J* = 6.0 Hz, 1H, H14), 6.446 (*d*, *J* = 6.5 Hz, 1H, H13), 4.607 (*t*, *J* = 3.3 Hz, 1H, H7), 3.435 (*d*, *J* = 8.5 Hz, 2H, H2, H12), 3.379 (*dd*, *J* = 8.3, 3.3 Hz, 2H, H6, H8); ^13^C NMR (126 MHz, CD_2_Cl_2_) δ 175.04 (C5, C9), 172.73 (C3, C11), 136.98 (C18), 135.21 (C16), 132.06 (C24, C30), 129.66 (C26, C28, C32, C34), 129.42 (C27, C33), 129.34 (C20, C22), 128.42 (C21), 127.71 (C17), 126.92 (C25, C29, C31, C35), 126.71 (C14), 126.63 (C19, C23), 126.16 (C13), 62.32 (C1), 47.35 (C2, C12), 41.72 (C6, C8), 40.47 (C7); IR (KBr, cm^−1^) 3065 (C—H), 2926 (C—H), 2853 (C—H), 1717 (C=O), 1497 (C=C), 1379 (C=C), 1188 (C—N), 743, 727; MS (ESI, PEG, *m/z*) [*M*+H]^+^ calculated for C_32_H_22_N_2_O_4_S 531.1373, found 531.1383.

Recrystallization from many solvent combinations was attempted. The first good crystals were obtained from toluene:1,2-di­chloro­ethane (DCE) [ratio 19:1]. These were empirically (3)·0.5C_7_H_8_·0.5DCE, with toluene on inversion centers and DCE on twofold axes; both solvents were disordered. Recrystallization from *p*-xylene gave orderly crystals of (3) by suction filtration after 5 days of slow evaporation at room temperature. No conditions were found that gave neat crystals of (3).

## Refinement   

Crystal data, data collection, and structure refinement details are summarized in Table 2 ▶. C-bound H atoms were placed in calculated positions and refined as riding atoms, with C—H = 0.0.95–0.98 Å and with *U*_iso_(H) = 1.5*U*_eq_(C) for methyl H atoms and = 1.2*U*_eq_(C) for other H atoms.

## Supplementary Material

Crystal structure: contains datablock(s) I, global. DOI: 10.1107/S1600536814025094/su5020sup1.cif

Structure factors: contains datablock(s) I. DOI: 10.1107/S1600536814025094/su5020Isup2.hkl

Supporting information file. DOI: 10.1107/S1600536814025094/su5020Isup3.cml

CCDC reference: 1034481

Additional supporting information:  crystallographic information; 3D view; checkCIF report

## Figures and Tables

**Figure 5:**
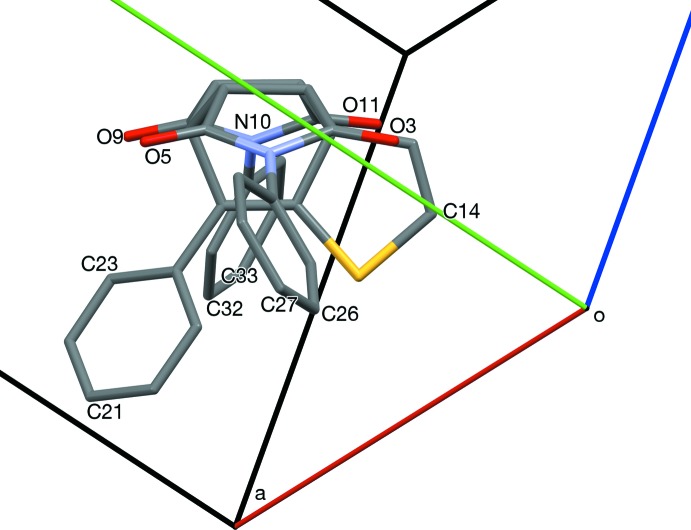
Twisting of *N*-phenyl rings (C27 forward, C33 behind) viewed along [514], normal to the thio­phene moiety.

**Figure 7:**
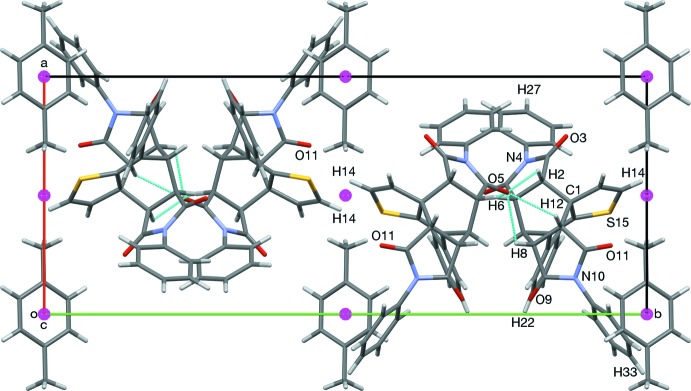
A view along the *c* axis of the crystal packing of compound (3). *p*-Xylene mol­ecules and inversion-related pairs (O11⋯H14) of mol­ecules occupy alternating layers about inversion centers.

**Table 1:** Hydrogen-bond geometry (, )

*D*H*A*	*D*H	H*A*	*D* *A*	*D*H*A*
C23H23O9	0.95	2.59	3.435(2)	149
C2H2O5^i^	1.00	2.46	3.158(2)	126
C6H6O5^i^	1.00	2.56	3.206(2)	122
C8H8O5^i^	1.00	2.66	3.269(2)	131
C12H12O5^i^	1.00	2.47	3.182(2)	128
C14H14O11^ii^	0.95	2.50	3.413(2)	162

Symmetry codes: (i) 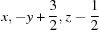; (ii) 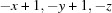.

**Table 2:** Experimental details

Crystal data	
Chemical formula	C_32_H_22_N_2_O_4_S0.5C_8_H_10_
*M*_r_	583.65
Crystal system, space group	Monoclinic, *P*2_1_/*c*
Temperature (K)	123
*a*, *b*, *c* ()	10.5944(14), 26.529(4), 10.4286(14)
()	99.675(2)
*V* (^3^)	2889.4(7)
*Z*	4
Radiation type	Mo *K*
(mm^1^)	0.16
Crystal size (mm)	0.45 0.22 0.22

Data collection	
Diffractometer	Bruker APEXII CCD
Absorption correction	Multi-scan (*SADABS*; Sheldrick, 1996 ▶)
*T*_min_, *T*_max_	0.685, 0.746
No. of measured, independent and observed [*I* > 2(*I*)] reflections	33190, 6576, 5803
*R*_int_	0.025
(sin /)_max_ (^1^)	0.648

Refinement	
*R*[*F* ^2^ > 2(*F* ^2^)], *wR*(*F* ^2^), *S*	0.040, 0.109, 1.00
No. of reflections	6576
No. of parameters	388
H-atom treatment	H-atom parameters constrained
_max_, _min_ (e ^3^)	0.38, 0.36

Computer programs: *APEX2* and *SAINT* (Bruker, 2007 ▶), *SHELXS97*, *SHELXL2014* and *SHELXTL2008* (Sheldrick, 2008 ▶), *Mercury* (Macrae *et al.*, 2008 ▶), *enCIFer* (Allen *et al.*, 2004 ▶) and *publCIF* (Westrip, 2010 ▶).

## supplementary crystallographic information

### Crystal data

**Table:**

C_32_H_22_N_2_O_4_S·0.5C_8_H_10_	*D*_x_ = 1.342 Mg m^−^^3^
*M**_r_* = 583.65	Melting point: 554 K
Monoclinic, *P*2_1_/*c*	Mo *K*α radiation, λ = 0.71073 Å
*a* = 10.5944 (14) Å	Cell parameters from 2771 reflections
*b* = 26.529 (4) Å	θ = 3.1–27.4°
*c* = 10.4286 (14) Å	µ = 0.16 mm^−^^1^
β = 99.675 (2)°	*T* = 123 K
*V* = 2889.4 (7) Å^3^	Plate, colourless
*Z* = 4	0.45 × 0.22 × 0.22 mm
*F*(000) = 1220	

### Data collection

**Table:**

Bruker APEXII CCD diffractometer	5803 reflections with *I* > 2σ(*I*)
Radiation source: sealed tube	*R*_int_ = 0.025
φ and ω scans	θ_max_ = 27.4°, θ_min_ = 2.0°
Absorption correction: multi-scan (*SADABS*; Sheldrick, 1996)	*h* = −13→13
*T*_min_ = 0.685, *T*_max_ = 0.746	*k* = −34→34
33190 measured reflections	*l* = −13→13
6576 independent reflections	

### Refinement

**Table:**

Refinement on *F*^2^	0 restraints
Least-squares matrix: full	Hydrogen site location: inferred from neighbouring sites
*R*[*F*^2^ > 2σ(*F*^2^)] = 0.040	H-atom parameters constrained
*wR*(*F*^2^) = 0.109	*w* = 1/[σ^2^(*F*_o_^2^) + (0.0547*P*)^2^ + 1.9057*P*] where *P* = (*F*_o_^2^ + 2*F*_c_^2^)/3
*S* = 1.00	(Δ/σ)_max_ = 0.001
6576 reflections	Δρ_max_ = 0.38 e Å^−^^3^
388 parameters	Δρ_min_ = −0.36 e Å^−^^3^

### Special details

**Table:**

Geometry. All e.s.d.'s (except the e.s.d. in the dihedral angle between two l.s. planes) are estimated using the full covariance matrix. The cell e.s.d.'s are taken into account individually in the estimation of e.s.d.'s in distances, angles and torsion angles; correlations between e.s.d.'s in cell parameters are only used when they are defined by crystal symmetry. An approximate (isotropic) treatment of cell e.s.d.'s is used for estimating e.s.d.'s involving l.s. planes.

### Fractional atomic coordinates and isotropic or equivalent isotropic displacement parameters (Å^2^)

**Table:**

	*x*	*y*	*z*	*U*_iso_*/*U*_eq_	
C1	0.47103 (13)	0.62662 (5)	0.07444 (13)	0.0168 (3)	
C2	0.56246 (13)	0.67315 (5)	0.10206 (13)	0.0171 (3)	
H2	0.6037	0.6805	0.0246	0.021*	
O3	0.74841 (10)	0.63409 (4)	0.23636 (11)	0.0275 (2)	
C3	0.66409 (13)	0.66474 (5)	0.22147 (13)	0.0188 (3)	
N4	0.64349 (11)	0.70008 (4)	0.31639 (11)	0.0183 (2)	
O5	0.51276 (10)	0.76804 (4)	0.33509 (10)	0.0237 (2)	
C5	0.54585 (13)	0.73378 (5)	0.27202 (13)	0.0174 (3)	
C6	0.48591 (13)	0.71923 (5)	0.13506 (13)	0.0168 (3)	
H6	0.4931	0.7475	0.0733	0.020*	
C7	0.34372 (13)	0.70480 (5)	0.13224 (13)	0.0171 (3)	
H7	0.2938	0.7341	0.1573	0.020*	
C8	0.29297 (13)	0.68806 (5)	−0.00915 (13)	0.0186 (3)	
H8	0.2980	0.7166	−0.0709	0.022*	
O9	0.06508 (10)	0.69309 (4)	0.00144 (11)	0.0270 (2)	
C9	0.15643 (13)	0.66936 (5)	−0.02085 (13)	0.0202 (3)	
N10	0.15215 (11)	0.61919 (4)	−0.06089 (11)	0.0195 (2)	
O11	0.28909 (10)	0.56023 (4)	−0.12507 (11)	0.0262 (2)	
C11	0.27107 (13)	0.60134 (5)	−0.08300 (13)	0.0196 (3)	
C12	0.36946 (13)	0.64258 (5)	−0.04520 (13)	0.0181 (3)	
H12	0.4127	0.6511	−0.1206	0.022*	
C13	0.53628 (13)	0.57771 (5)	0.05823 (14)	0.0215 (3)	
H13	0.5845	0.5721	−0.0095	0.026*	
C14	0.52206 (14)	0.54274 (6)	0.14507 (15)	0.0246 (3)	
H14	0.5584	0.5101	0.1437	0.030*	
S15	0.43063 (4)	0.56125 (2)	0.26360 (4)	0.02309 (10)	
C16	0.40457 (13)	0.62075 (5)	0.19169 (13)	0.0166 (3)	
C17	0.33937 (12)	0.66069 (5)	0.22472 (13)	0.0168 (3)	
C18	0.27549 (13)	0.66453 (5)	0.34017 (13)	0.0186 (3)	
C19	0.33888 (16)	0.64756 (7)	0.46108 (15)	0.0296 (3)	
H19	0.4224	0.6336	0.4683	0.035*	
C20	0.28055 (19)	0.65094 (8)	0.57056 (17)	0.0400 (4)	
H20	0.3243	0.6393	0.6522	0.048*	
C21	0.15919 (18)	0.67111 (7)	0.56115 (18)	0.0378 (4)	
H21	0.1191	0.6731	0.6359	0.045*	
C22	0.09641 (16)	0.68835 (7)	0.44274 (18)	0.0332 (4)	
H22	0.0127	0.7021	0.4361	0.040*	
C23	0.15467 (15)	0.68579 (6)	0.33309 (16)	0.0262 (3)	
H23	0.1115	0.6987	0.2526	0.031*	
C24	0.71510 (13)	0.70018 (6)	0.44597 (13)	0.0202 (3)	
C25	0.71973 (16)	0.65636 (7)	0.51885 (16)	0.0303 (3)	
H25	0.6760	0.6269	0.4835	0.036*	
C26	0.78922 (18)	0.65597 (8)	0.64436 (17)	0.0413 (4)	
H26	0.7930	0.6262	0.6953	0.050*	
C27	0.85297 (16)	0.69899 (8)	0.69534 (16)	0.0401 (5)	
H27	0.9007	0.6985	0.7810	0.048*	
C28	0.84726 (15)	0.74241 (8)	0.62212 (16)	0.0344 (4)	
H28	0.8905	0.7719	0.6579	0.041*	
C29	0.77860 (13)	0.74341 (6)	0.49609 (15)	0.0244 (3)	
H29	0.7753	0.7732	0.4452	0.029*	
C30	0.03938 (13)	0.58848 (5)	−0.06870 (14)	0.0201 (3)	
C31	−0.02016 (15)	0.58564 (6)	0.03952 (15)	0.0261 (3)	
H31	0.0120	0.6044	0.1156	0.031*	
C32	−0.12704 (15)	0.55524 (6)	0.03589 (17)	0.0299 (3)	
H32	−0.1686	0.5531	0.1096	0.036*	
C33	−0.17302 (15)	0.52803 (6)	−0.07528 (17)	0.0284 (3)	
H33	−0.2459	0.5070	−0.0776	0.034*	
C34	−0.11321 (15)	0.53137 (6)	−0.18289 (16)	0.0288 (3)	
H34	−0.1456	0.5127	−0.2591	0.035*	
C35	−0.00598 (15)	0.56179 (6)	−0.18087 (15)	0.0247 (3)	
H35	0.0352	0.5642	−0.2549	0.030*	
C36	−0.0645 (2)	0.53975 (8)	0.43607 (17)	0.0449 (5)	
H36	−0.1088	0.5675	0.3918	0.054*	
C37	0.0665 (2)	0.54081 (8)	0.46333 (18)	0.0451 (5)	
H37	0.1114	0.5690	0.4375	0.054*	
C38	0.1347 (2)	0.50084 (8)	0.52868 (17)	0.0449 (5)	
C39	0.2786 (2)	0.50191 (11)	0.5586 (2)	0.0633 (7)	
H39A	0.3098	0.5334	0.5262	0.095*	
H39B	0.3067	0.4997	0.6529	0.095*	
H39C	0.3131	0.4733	0.5163	0.095*	

### Atomic displacement parameters (Å^2^)

**Table:**

	*U*^11^	*U*^22^	*U*^33^	*U*^12^	*U*^13^	*U*^23^
S15	0.0287 (2)	0.01758 (17)	0.02321 (18)	0.00367 (13)	0.00515 (14)	0.00323 (13)
O3	0.0221 (5)	0.0302 (6)	0.0284 (6)	0.0087 (4)	−0.0012 (4)	−0.0075 (4)
O5	0.0243 (5)	0.0242 (5)	0.0216 (5)	0.0044 (4)	0.0006 (4)	−0.0065 (4)
O9	0.0203 (5)	0.0227 (5)	0.0357 (6)	0.0046 (4)	−0.0021 (4)	−0.0033 (4)
O11	0.0239 (5)	0.0247 (5)	0.0301 (6)	−0.0002 (4)	0.0047 (4)	−0.0097 (4)
N4	0.0175 (5)	0.0207 (6)	0.0162 (5)	0.0013 (4)	0.0013 (4)	−0.0022 (4)
N10	0.0186 (6)	0.0191 (6)	0.0198 (6)	0.0000 (4)	0.0000 (4)	−0.0019 (4)
C1	0.0176 (6)	0.0177 (6)	0.0150 (6)	0.0006 (5)	0.0022 (5)	−0.0018 (5)
C2	0.0174 (6)	0.0183 (6)	0.0159 (6)	0.0003 (5)	0.0034 (5)	−0.0014 (5)
C3	0.0174 (6)	0.0210 (7)	0.0181 (6)	0.0002 (5)	0.0034 (5)	−0.0027 (5)
C5	0.0164 (6)	0.0186 (6)	0.0173 (6)	−0.0013 (5)	0.0026 (5)	−0.0002 (5)
C6	0.0181 (6)	0.0162 (6)	0.0159 (6)	0.0000 (5)	0.0024 (5)	−0.0003 (5)
C7	0.0169 (6)	0.0164 (6)	0.0170 (6)	0.0015 (5)	0.0003 (5)	−0.0004 (5)
C8	0.0197 (7)	0.0177 (6)	0.0169 (6)	0.0007 (5)	−0.0014 (5)	0.0014 (5)
C9	0.0208 (7)	0.0199 (7)	0.0177 (6)	0.0017 (5)	−0.0031 (5)	0.0010 (5)
C11	0.0202 (7)	0.0236 (7)	0.0146 (6)	−0.0002 (5)	0.0014 (5)	−0.0008 (5)
C12	0.0205 (6)	0.0193 (6)	0.0141 (6)	−0.0002 (5)	0.0016 (5)	−0.0008 (5)
C13	0.0193 (7)	0.0233 (7)	0.0214 (7)	0.0032 (5)	0.0025 (5)	−0.0059 (5)
C14	0.0254 (7)	0.0206 (7)	0.0268 (7)	0.0061 (6)	0.0013 (6)	−0.0054 (6)
C16	0.0168 (6)	0.0174 (6)	0.0147 (6)	−0.0006 (5)	0.0004 (5)	0.0012 (5)
C17	0.0151 (6)	0.0187 (6)	0.0161 (6)	−0.0007 (5)	0.0011 (5)	−0.0001 (5)
C18	0.0207 (7)	0.0172 (6)	0.0196 (6)	0.0010 (5)	0.0085 (5)	−0.0019 (5)
C19	0.0259 (8)	0.0418 (9)	0.0214 (7)	0.0042 (7)	0.0052 (6)	−0.0001 (6)
C20	0.0424 (10)	0.0570 (12)	0.0221 (8)	0.0027 (9)	0.0095 (7)	−0.0010 (8)
C21	0.0425 (10)	0.0432 (10)	0.0336 (9)	−0.0048 (8)	0.0231 (8)	−0.0092 (7)
C22	0.0270 (8)	0.0328 (9)	0.0434 (10)	0.0008 (7)	0.0167 (7)	−0.0083 (7)
C23	0.0234 (7)	0.0256 (7)	0.0306 (8)	0.0031 (6)	0.0076 (6)	−0.0033 (6)
C24	0.0147 (6)	0.0295 (7)	0.0160 (6)	0.0036 (5)	0.0016 (5)	−0.0030 (5)
C25	0.0306 (8)	0.0330 (8)	0.0261 (8)	0.0064 (7)	0.0014 (6)	0.0023 (6)
C26	0.0407 (10)	0.0557 (12)	0.0258 (8)	0.0200 (9)	0.0012 (7)	0.0096 (8)
C27	0.0257 (8)	0.0732 (14)	0.0190 (7)	0.0187 (8)	−0.0037 (6)	−0.0078 (8)
C28	0.0160 (7)	0.0587 (11)	0.0277 (8)	0.0028 (7)	0.0009 (6)	−0.0198 (8)
C29	0.0161 (6)	0.0345 (8)	0.0232 (7)	0.0002 (6)	0.0044 (5)	−0.0076 (6)
C30	0.0173 (6)	0.0183 (6)	0.0234 (7)	0.0013 (5)	−0.0006 (5)	−0.0011 (5)
C31	0.0245 (7)	0.0279 (8)	0.0256 (7)	−0.0005 (6)	0.0036 (6)	−0.0059 (6)
C32	0.0253 (8)	0.0324 (8)	0.0339 (8)	−0.0010 (6)	0.0103 (6)	−0.0038 (7)
C33	0.0202 (7)	0.0230 (7)	0.0416 (9)	−0.0018 (6)	0.0037 (6)	−0.0036 (6)
C34	0.0266 (8)	0.0259 (8)	0.0316 (8)	−0.0029 (6)	−0.0017 (6)	−0.0077 (6)
C35	0.0259 (7)	0.0253 (7)	0.0219 (7)	−0.0006 (6)	0.0012 (6)	−0.0033 (6)
C36	0.0680 (14)	0.0399 (10)	0.0260 (8)	0.0267 (10)	0.0057 (8)	0.0051 (7)
C37	0.0680 (14)	0.0381 (10)	0.0294 (9)	0.0164 (9)	0.0082 (9)	0.0007 (8)
C38	0.0593 (12)	0.0504 (11)	0.0240 (8)	0.0221 (10)	0.0037 (8)	−0.0016 (8)
C39	0.0584 (14)	0.0869 (19)	0.0425 (12)	0.0185 (13)	0.0027 (10)	0.0007 (12)

### Geometric parameters (Å, º)

**Table:**

S15—C14	1.7635 (16)	C20—C21	1.381 (3)
S15—C16	1.7495 (14)	C20—H20	0.9500
O3—C3	1.1986 (17)	C21—C22	1.379 (3)
O5—C5	1.2081 (17)	C21—H21	0.9500
O9—C9	1.2094 (18)	C22—C23	1.389 (2)
O11—C11	1.2028 (17)	C22—H22	0.9500
N4—C3	1.4069 (17)	C23—H23	0.9500
N4—C5	1.3865 (17)	C24—C25	1.385 (2)
N4—C24	1.4342 (17)	C24—C29	1.387 (2)
N10—C9	1.3934 (18)	C25—C26	1.390 (2)
N10—C11	1.4008 (18)	C25—H25	0.9500
N10—C30	1.4370 (18)	C26—C27	1.386 (3)
C1—C2	1.5655 (18)	C26—H26	0.9500
C1—C12	1.5629 (18)	C27—C28	1.378 (3)
C1—C13	1.4930 (19)	C27—H27	0.9500
C1—C16	1.5181 (18)	C28—C29	1.391 (2)
C2—C3	1.5196 (18)	C28—H28	0.9500
C2—C6	1.5377 (18)	C29—H29	0.9500
C2—H2	1.0000	C30—C31	1.384 (2)
C5—C6	1.5124 (18)	C30—C35	1.383 (2)
C6—C7	1.5498 (18)	C31—C32	1.386 (2)
C6—H6	1.0000	C31—H31	0.9500
C7—C8	1.5472 (18)	C32—C33	1.383 (2)
C7—C17	1.5220 (18)	C32—H32	0.9500
C7—H7	1.0000	C33—C34	1.381 (2)
C8—C9	1.5147 (19)	C33—H33	0.9500
C8—C12	1.5351 (19)	C34—C35	1.391 (2)
C8—H8	1.0000	C34—H34	0.9500
C11—C12	1.5167 (19)	C35—H35	0.9500
C12—H12	1.0000	C36—C37	1.370 (3)
C13—C14	1.323 (2)	C36—C38^i^	1.393 (3)
C13—H13	0.9500	C36—H36	0.9500
C14—H14	0.9500	C37—C38	1.395 (3)
C16—C17	1.3410 (19)	C37—H37	0.9500
C17—C18	1.4805 (18)	C38—C39	1.504 (3)
C18—C19	1.400 (2)	C39—H39A	0.9800
C18—C23	1.389 (2)	C39—H39B	0.9800
C19—C20	1.390 (2)	C39—H39C	0.9800
C19—H19	0.9500		
			
C14—S15—C16	90.95 (7)	C17—C18—C23	121.85 (13)
C3—N4—C5	112.96 (11)	C19—C18—C23	118.48 (13)
C3—N4—C24	122.96 (11)	C18—C19—C20	120.47 (15)
C5—N4—C24	124.07 (11)	C18—C19—H19	119.8
C9—N10—C11	112.78 (12)	C20—C19—H19	119.8
C9—N10—C30	122.91 (12)	C19—C20—C21	120.27 (17)
C11—N10—C30	124.15 (12)	C19—C20—H20	119.9
C2—C1—C12	104.78 (10)	C21—C20—H20	119.9
C2—C1—C13	114.97 (11)	C20—C21—C22	119.70 (15)
C2—C1—C16	106.80 (10)	C20—C21—H21	120.1
C12—C1—C13	114.50 (11)	C22—C21—H21	120.1
C12—C1—C16	108.69 (11)	C21—C22—C23	120.46 (16)
C13—C1—C16	106.76 (11)	C21—C22—H22	119.8
C1—C2—C3	111.49 (11)	C23—C22—H22	119.8
C1—C2—C6	109.56 (11)	C18—C23—C22	120.58 (15)
C3—C2—C6	105.13 (10)	C18—C23—H23	119.7
C1—C2—H2	110.2	C22—C23—H23	119.7
C3—C2—H2	110.2	N4—C24—C25	118.68 (13)
C6—C2—H2	110.2	N4—C24—C29	120.19 (13)
O3—C3—N4	124.15 (13)	C25—C24—C29	121.13 (14)
O3—C3—C2	128.01 (13)	C24—C25—C26	119.17 (17)
N4—C3—C2	107.84 (11)	C24—C25—H25	120.4
O5—C5—N4	124.88 (12)	C26—C25—H25	120.4
O5—C5—C6	126.30 (12)	C25—C26—C27	120.14 (18)
N4—C5—C6	108.79 (11)	C25—C26—H26	119.9
C2—C6—C5	105.11 (11)	C27—C26—H26	119.9
C2—C6—C7	110.30 (11)	C26—C27—C28	120.18 (15)
C5—C6—C7	109.48 (11)	C26—C27—H27	119.9
C2—C6—H6	110.6	C28—C27—H27	119.9
C5—C6—H6	110.6	C27—C28—C29	120.45 (17)
C7—C6—H6	110.6	C27—C28—H28	119.8
C6—C7—C8	105.66 (11)	C29—C28—H28	119.8
C6—C7—C17	108.10 (10)	C24—C29—C28	118.93 (16)
C8—C7—C17	109.92 (11)	C24—C29—H29	120.5
C6—C7—H7	111.0	C28—C29—H29	120.5
C8—C7—H7	111.0	N10—C30—C31	118.24 (12)
C17—C7—H7	111.0	N10—C30—C35	120.38 (13)
C7—C8—C9	110.18 (11)	C35—C30—C31	121.37 (14)
C7—C8—C12	110.23 (11)	C30—C31—C32	119.45 (14)
C9—C8—C12	105.01 (11)	C30—C31—H31	120.3
C7—C8—H8	110.4	C32—C31—H31	120.3
C9—C8—H8	110.4	C31—C32—C32	119.85 (15)
C12—C8—H8	110.4	C31—C32—H32	120.1
O9—C9—N10	124.61 (13)	C33—C32—H32	120.1
O9—C9—C8	126.75 (13)	C32—C33—C34	120.17 (14)
N10—C9—C8	108.64 (12)	C32—C33—H33	119.9
O11—C11—N10	124.66 (13)	C34—C33—H33	119.9
O11—C11—C12	127.20 (13)	C33—C34—C35	120.67 (14)
N10—C11—C12	108.14 (11)	C33—C34—H34	119.7
C1—C12—C8	109.78 (10)	C35—C34—H34	119.7
C1—C12—C11	111.29 (11)	C30—C35—C34	118.48 (14)
C8—C12—C11	105.22 (11)	C30—C35—H35	120.8
C1—C12—H12	110.1	C34—C35—H35	120.8
C8—C12—H12	110.1	C37—C36—C38^i^	121.92 (18)
C11—C12—H12	110.1	C37—C36—H36	119.0
C1—C13—C14	115.08 (13)	C38^i^—C36—H36	119.0
C1—C13—H13	122.5	C36—C37—C38	120.6 (2)
C14—C13—H13	122.5	C36—C37—H37	119.7
S15—C14—C13	115.07 (11)	C38—C37—H37	119.7
S15—C14—H14	122.5	C36^i^—C38—C37	117.4 (2)
C13—C14—H14	122.5	C36^i^—C38—C39	121.83 (19)
S15—C16—C1	112.12 (9)	C37—C38—C39	120.7 (2)
S15—C16—C17	130.82 (11)	C38—C39—H39A	109.5
C1—C16—C17	117.04 (12)	C38—C39—H39B	109.5
C7—C17—C16	111.89 (12)	C38—C39—H39C	109.5
C7—C17—C18	121.95 (12)	H39A—C39—H39B	109.5
C16—C17—C18	126.07 (12)	H39A—C39—H39C	109.5
C17—C18—C19	119.66 (13)	H39B—C39—H39C	109.5
			
S15—C14—C13—C1	−0.77 (17)	C5—C6—C7—C17	−59.61 (13)
S15—C16—C1—C2	−122.42 (10)	C6—C2—C1—C12	61.10 (13)
S15—C16—C1—C12	125.04 (10)	C6—C2—C1—C13	−172.32 (11)
S15—C16—C1—C13	1.04 (13)	C6—C2—C1—C16	−54.10 (13)
S15—C16—C17—C7	179.78 (10)	C6—C5—N4—C24	−175.22 (12)
S15—C16—C17—C18	3.1 (2)	C6—C7—C8—C9	175.86 (11)
O3—C3—N4—C5	175.46 (14)	C6—C7—C8—C12	60.43 (14)
O3—C3—N4—C24	−5.7 (2)	C6—C7—C17—C16	−58.81 (14)
O3—C3—C2—C1	64.82 (19)	C6—C7—C17—C18	118.02 (13)
O3—C3—C2—C6	−176.55 (15)	C7—C8—C12—C11	121.82 (12)
O5—C5—N4—C3	−178.39 (13)	C7—C17—C18—C19	−132.20 (14)
O5—C5—N4—C24	2.8 (2)	C7—C17—C18—C23	46.14 (19)
O5—C5—C6—C2	−179.22 (13)	C8—C7—C17—C16	56.06 (15)
O5—C5—C6—C7	−60.75 (18)	C8—C7—C17—C18	−127.11 (13)
O9—C9—N10—C11	178.47 (14)	C8—C9—N10—C11	−2.16 (15)
O9—C9—N10—C30	−5.9 (2)	C8—C9—N10—C30	173.45 (12)
O9—C9—C8—C7	59.85 (18)	C8—C12—C1—C13	170.30 (12)
O9—C9—C8—C12	178.53 (14)	C8—C12—C1—C16	51.04 (14)
O11—C11—N10—C9	−175.58 (14)	C9—N10—C11—C12	4.28 (15)
O11—C11—N10—C30	8.9 (2)	C9—N10—C30—C31	−52.23 (19)
O11—C11—C12—C1	−65.82 (18)	C9—N10—C30—C35	129.14 (15)
O11—C11—C12—C8	175.34 (14)	C9—C8—C12—C11	3.17 (13)
N4—C3—C2—C1	−115.29 (12)	C9—C8—C7—C17	59.45 (14)
N4—C3—C2—C6	3.34 (14)	C11—N10—C30—C31	122.88 (15)
N4—C5—C6—C2	−1.26 (14)	C11—N10—C30—C35	−55.75 (19)
N4—C5—C6—C7	117.21 (12)	C11—C12—C1—C13	54.22 (15)
N4—C24—C25—C26	179.77 (14)	C11—C12—C1—C16	−65.04 (14)
N4—C24—C29—C28	179.97 (13)	C12—C1—C13—C14	−120.51 (14)
N10—C9—C8—C7	−119.51 (12)	C12—C1—C16—C17	−56.16 (15)
N10—C9—C8—C12	−0.83 (14)	C12—C11—N10—C30	−171.27 (12)
N10—C11—C12—C1	114.33 (12)	C12—C8—C7—C17	−55.98 (14)
N10—C11—C12—C8	−4.51 (14)	C13—C1—C16—C17	179.84 (12)
N10—C30—C31—C32	−178.26 (14)	C13—C14—S15—C16	1.19 (13)
N10—C30—C35—C34	178.12 (13)	C14—S15—C16—C17	−179.83 (14)
C1—C2—C6—C5	118.66 (11)	C14—C13—C1—C16	−0.18 (17)
C1—C2—C6—C7	0.75 (14)	C16—C17—C18—C19	44.2 (2)
C1—C12—C8—C7	1.97 (15)	C16—C17—C18—C23	−137.49 (15)
C1—C12—C8—C9	−116.67 (12)	C17—C18—C19—C20	179.87 (16)
C1—C16—S15—C14	−1.25 (10)	C17—C18—C23—C22	179.23 (14)
C1—C16—C17—C7	1.26 (17)	C18—C19—C20—C21	0.0 (3)
C1—C16—C17—C18	−175.42 (12)	C18—C23—C22—C21	1.9 (2)
C2—C1—C13—C14	118.06 (14)	C19—C18—C23—C22	−2.4 (2)
C2—C1—C16—C17	56.38 (15)	C19—C20—C21—C22	−0.6 (3)
C2—C1—C12—C8	−62.83 (13)	C20—C19—C18—C23	1.5 (2)
C2—C1—C12—C11	−178.92 (11)	C20—C21—C22—C23	−0.3 (3)
C2—C3—N4—C5	−4.44 (15)	C24—C25—C26—C27	−0.1 (3)
C2—C3—N4—C24	174.41 (12)	C24—C29—C28—C27	0.6 (2)
C2—C6—C7—C8	−62.06 (13)	C25—C24—C29—C28	−0.4 (2)
C2—C6—C7—C17	55.57 (14)	C25—C26—C27—C28	0.3 (3)
C3—N4—C5—C6	3.62 (15)	C26—C25—C24—C29	0.1 (2)
C3—N4—C24—C25	−54.29 (19)	C26—C27—C28—C29	−0.6 (2)
C3—N4—C24—C29	125.40 (15)	C30—C31—C32—C33	0.1 (2)
C3—C2—C1—C12	177.05 (11)	C30—C35—C34—C33	0.1 (2)
C3—C2—C1—C13	−56.37 (15)	C31—C30—C35—C34	−0.5 (2)
C3—C2—C1—C16	61.85 (13)	C31—C32—C33—C34	−0.4 (2)
C3—C2—C6—C5	−1.26 (13)	C32—C31—C30—C35	0.4 (2)
C3—C2—C6—C7	−119.17 (11)	C32—C33—C34—C35	0.3 (2)
C5—N4—C24—C25	124.43 (15)	C36—C37—C38—C36^i^	0.3 (3)
C5—N4—C24—C29	−55.88 (19)	C36—C37—C38—C39	179.96 (19)
C5—C6—C7—C8	−177.24 (11)	C38^i^—C36—C37—C38	−0.3 (3)

Symmetry code: (i) −*x*, −*y*+1, −*z*+1.

### Hydrogen-bond geometry (Å, º)

**Table:**

*D*—H···*A*	*D*—H	H···*A*	*D*···*A*	*D*—H···*A*
C23—H23···O9	0.95	2.59	3.435 (2)	149
C2—H2···O5^ii^	1.00	2.46	3.158 (2)	126
C6—H6···O5^ii^	1.00	2.56	3.206 (2)	122
C8—H8···O5^ii^	1.00	2.66	3.269 (2)	131
C12—H12···O5^ii^	1.00	2.47	3.182 (2)	128
C14—H14···O11^iii^	0.95	2.50	3.413 (2)	162

Symmetry codes: (ii) *x*, −*y*+3/2, *z*−1/2; (iii) −*x*+1, −*y*+1, −*z*.
